# High-efficiency flexible organic solar cells with a polymer-incorporated pseudo-planar heterojunction

**DOI:** 10.1186/s11671-024-03982-1

**Published:** 2024-03-04

**Authors:** Lin Zhang, Yuxin He, Wen Deng, Xueliang Guo, Zhaozhao Bi, Jie Zeng, Hui Huang, Guangye Zhang, Chen Xie, Yong Zhang, Xiaotian Hu, Wei Ma, Yongbo Yuan, Xiaoming Yuan

**Affiliations:** 1 Hunan Key Laboratory for Super Microstructure and Ultrafast Process, School of Physics, Central South University, Changsha, 410083 China; 2 State Key Laboratory for Mechanical Behavior of Materials, Xi’an Jiaotong University, Xi’an, 710049 China; 3 Institute of Polymers and Energy Chemistry, College of Chemistry and Chemical Engineering, Nanchang University, Nanchang, 330031 China; 4 Department of Materials Science and Engineering, and Shenzhen Engineering Research and Development Center for Flexible Solar Cells, Southern University of Science and Technology, Shenzhen, 518055 China; 5 College of New Materials and New Energies, Shenzhen Technology University, Shenzhen, 518118 China

**Keywords:** Organic solar cells, Flexible, Blade-coating, Non-halogenated solvent, Vertical component distribution

## Abstract

Organic solar cells (OSCs) are considered as a crucial energy source for flexible and wearable electronics. Pseudo-planar heterojunction (PPHJ) OSCs simplify the solution preparation and morphology control. However, non-halogenated solvent-printed PPHJ often have an undesirable vertical component distribution and insufficient donor/acceptor interfaces. Additionally, the inherent brittleness of non-fullerene small molecule acceptors (NFSMAs) in PPHJ leads to poor flexibility, and the NFSMAs solution shows inadequate viscosity during the printing of acceptor layer. Herein, we propose a novel approach termed polymer-incorporated pseudo-planar heterojunction (PiPPHJ), wherein a small amount of polymer donor is introduced into the NFSMAs layer. Our findings demonstrate that the incorporation of polymer increases the viscosity of acceptor solution, thereby improving the blade-coating processability and overall film quality. Simultaneously, this strategy effectively modulates the vertical component distribution, resulting in more donor/acceptor interfaces and an improved power conversion efficiency of 17.26%. Furthermore, PiPPHJ-based films exhibit superior tensile properties, with a crack onset strain of 12.0%, surpassing PPHJ-based films (9.6%). Consequently, large-area (1 cm^2^) flexible devices achieve a considerable efficiency of 13.30% and maintain excellent mechanical flexibility with 82% of the initial efficiency after 1000 bending cycles. These findings underscore the significant potential of PiPPHJ-based OSCs in flexible and wearable electronics.

**Supplementary Information:**

The online version contains supplementary material available at 10.1186/s11671-024-03982-1.

## Introduction

The organic solar cells (OSCs), with advantages such as light weight, flexibility and solution processability, have emerged as one of the most promising photovoltaic technologies for wearable electronics and building integration [1–4]. Considerable efforts have been devoted to enhancing the photovoltaic performance, boosting the power conversion efficiency (PCE) to over 19% [5–7]. Presently, most state-of-the art OSCs are fabricated by blend-casting (BC) with bulk heterojunction (BHJ) [8]. However, achieving precise regulation of nanoscale morphology in BHJ-based blend films relies excessively on factors like solvent selection, donor/acceptor (D/A) ratios, and solution concentration, leading to a complex control of the active layer morphology [9, 10]. The pseudo-planar heterojunction (PPHJ), obtained by sequential-casting (SC) with the separately dissolved donor and acceptor materials, can effectively address these challenges [11–13]. Nevertheless, the active layer based on PPHJ often exhibit an undesirable vertical component distribution and insufficient D/A interfaces, leading to low current density and poor photovoltaic performance [14]. This issue is exacerbated in the non-halogenated solvent-processed PPHJ OSCs due to the poor solubility of organic materials in non-halogenated solvents and significant solubility difference between the polymer donor and small molecule acceptor [15]. Therefore, for the non-halogenated solvent-processed PPHJ OSCs, optimizing the active layer morphology with a favorable vertical component distribution becomes essential [16].

Proverbially, the most distinctive advantage of OSCs is their flexibility, and the majority of highly efficient OSCs are prepared using polymer donors and non-fullerene small molecule acceptors (NFSMAs) [17, 18]. However, the inherent brittleness of NFSMAs enriched in the upper layer degrades the mechanical flexibility of PPHJ-based active layers [19]. Additionally, the solution properties of inks play a crucial role in film processing and are closely related to the final film quality [20, 21]. In large-area printing OSCs, such as blade-coating, solution viscosity emerges as a critical factor in influencing the processing performance and the nanomorphology of the obtained active layer [22, 23]. The solution must maintain an appropriate viscosity to ensure optimal processing. However, during the printing process of large-area films, NFSMAs often exhibit insufficient viscosity, particularly in non-halogenated solvents, limiting the processing window and resulting in suboptimal photovoltaic performance with low efficiency reproducibility for PPHJ-based OSCs [24]. Therefore, in non-halogenated solvent-printed PPHJ films, the optimization of acceptor solution viscosity, vertical component distribution for the active layer, and film flexibility should be simultaneously addressed to achieve highly efficient flexible OSCs with excellent reproducibility.

In this study, we proposed a novel approach termed polymer-incorporated pseudo-planar heterojunction (PiPPHJ), wherein a small amount of polymer donor (PM6) was introduced into the NFSMAs (BTP-eC9) layer in the non-halogenated solvent-printed OSCs. The incorporation of polymer increased the viscosity of the acceptor solution, effectively regulated the vertical component distribution and improved the mechanical tensile properties of active layer. As a result, the PiPPHJ-based OSCs exhibited an outstanding PCE of 17.26% with excellent reproducibility. Furthermore, large-area flexible OSCs were also fabricated, achieving a remarkable PCE of 13.30% and significant mechanical flexibility with 82% of the initial PCE after 1000 bending cycles.

## Experimental

### Fabrication of OSCs

Organic solar cell devices were fabricated in the conventional structure of ITO/PEDOT: PSS/Active layer/PDINN/Ag. The substrates were firstly cleaned using detergent, deionized water, acetone, and isopropanol for every 30 min, and then treated in ultraviolet ozone for 20 min. Followed by spin coating a thin layer (~ 30 nm) of PEDOT:PSS (Bayer baytron 4083) on the precleaned ITO-coated glass substrates at 4000 rpm for 30 s and then annealed at 150 °C for 15 min. Then, the PM6 solution was prepared in o-xylene at 10 mg mL^−1^ at 60 °C stirring. BTP-eC9 solution was prepared in o-xylene at 10 mg mL^−1^ at 80 °C stirring. The active layers are prepared by one-step blade-coating at 70 °C (base plate) under an ambient condition (40–70% RH). For the PiPPHJ-based devices, a PM6 solution is blade-coated on the PEDOT:PSS layer to form the lower layer at the blade speed of 45 mm/s, and subsequently, the BTP-eC9 solution with 0.2% DIO is blade-coated at 70 °C to form the upper layer at the blade speed of 60 mm/s. In order to introduce a certain proportion of polymer in the receptor layer, according to the actual volume capacity of the receptor BTP-eC9, the corresponding percentage of PM6 solution was added, and the upper layer of the receptor film was prepared after full mixing at 80 °C. Post-annealing treatment is also not required during the preparation of the entire active layer film. A thin cathode interface layer of PDINN is deposited via spin-coating its methanol solution (1 mg mL^−1^) at 3000 rpm for 30 s. Finally, the 100 nm thick Ag cathode is thermally deposited with a shadow mask in a vacuum of 1 × 10^−5^ Pa.

### Characterization

The current density–voltage (*J–V*) characteristics were measured in the glovebox with a Keithley 2400 measure unit under 1 sun, AM 1.5 G spectra (100 mW cm^−2^) from a solar simulator (S5-F5-3A Enli Tech. Co., Ltd., Taiwan). The light intensity was calibrated with a 20 mm × 20 mm monocrystalline silicon reference cell with KG5 filter (Enli Tech. Co., Ltd., Taiwan). The *J–V* curves are measured along the forward scan direction from − 0.2 to 1 V, with a scan step of 20 mV with delay time of 1 ms. The EQE was measured by solar cell spectral response measurement system QE-R3018 (Enli Tech. Co., Ltd., Taiwan). The light intensity at each wavelength was calibrated with a standard single-crystal Si photovoltaic cell.The film thickness was measured by a surface profilometer (Dektak XT, Bruker). The UV–Vis absorption spectrum was measured by a Shimadzu UV-3600 Plus Spectrophotometer.The steady PL spectra were measured by the FLS 920 (Edinburgh Instruments, Ltd) with excitation at 532 nm. AFM images were captured by using a Bruker Multimode-8 microscope system in the tapping mode under ambient conditions.

## Results and discussion

**Fig. 1:**
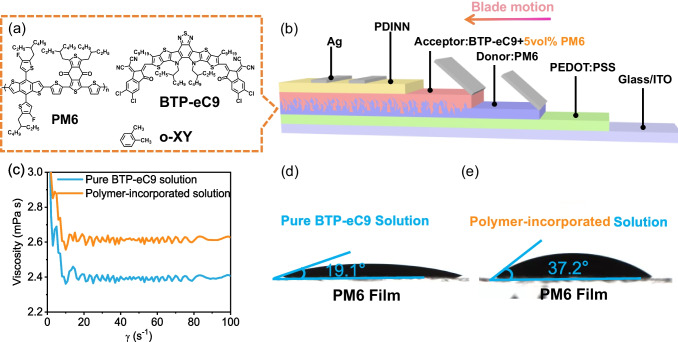
**a** Chemical structure of PM6, BTP-eC9 and o-XY. **b** The diagrammatic drawing of SC blade-coating and device architecture of OSCs. **c** Dependence of viscosity for the pure BTP-eC9 solution and polymer-incorporated solution on the shear rate. The contact angles of **d** pure BTP-eC9 solution and **e** polymer-incorporated solution on PM6 film

The chemical structures of the polymer donor PM6, non-fullerene small molecule acceptor BTP-eC9 and the non-halogenated solvent o-xylene (o-XY) are shown in Fig. 1a [25, 26]. A conventional structure of ITO/ PEDOT:PSS /active layer/PDINN/Ag was adopted in this work, and the diagrammatic drawing of sequential-casting with blade-coating is displayed in Fig. 1b. Rheometer studies were conducted to investigate the fluid mechanical properties of the precursor solution for the upper acceptor layer. As shown in Fig. 1c, the incorporation of PM6 polymer into acceptor solution increases the solution viscosity, which makes it suitable for solution shear coating and improves the blade-coating processability. Additionally, the wettability of the acceptor solution on PM6 film was studied by measuring the contact angle (Fig. 1d–e). The addition of polymer increases the contact angle from 19.1° to 37.2°, suggesting the larger adjustable range of the height and angle between blade and substrate, which broadens the processing window of blade-coating.

**Fig. 2:**
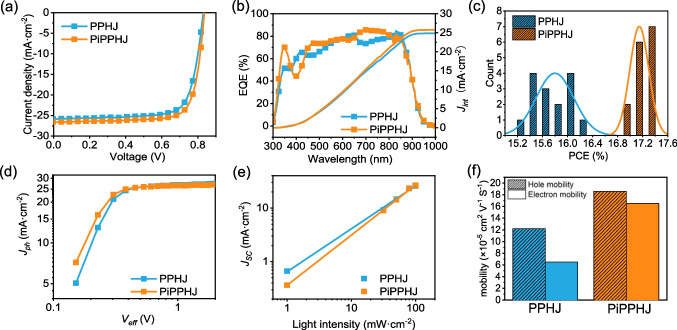
**a** *J-V* curves, **b** EQE spectra and **c** histograms of the PCE counts for the PPHJ-based and PiPPHJ-based devices. **d** *J*_*ph *_*− V*_*eff*_ curves and **e** the dependence of *J*_*SC*_ on light intensity for the devices based on PPHJ and PiPPHJ. **f** Histogram of carrier mobility

The OSCs (0.04 cm^2^) based on PPHJ and PiPPHJ with various PM6 contents were fabricated by blade-coating in ambient environment. As shown in Fig. 2a, S1 and Table 1, S1, the PPHJ-based device exhibited a PCE of 16.19%, while the optimized PiPPHJ-based device (5% PM6 content) achieved a superior PCE of 17.26%, with a *V*_*OC*_ of 0.836 V, *J*_*SC*_ of 26.61 mA cm^−2^, and FF of 77.57%. The *J*_*int*_ from external quantum efficiency (EQE) curves (Fig. 2b) matched well with the *J*_SC_ obtained from *J–V* curves[27]. To control variables and eliminate the influence of additives on device performance, the additive-free devices based on PPHJ and PiPPHJ were fabricated for comparison (Table S2), where it further underscores the positive impact of the PiPPHJ strategy on device performance. Notably, due to the improvement of film-forming performance, the PiPPHJ-based devices exhibit better film uniformity and superior reproducibility of photovoltaic efficiency (Fig. 2c).

**Table 1:** Photovoltaic parameters of non-halogenated solvent-printed OSCs under AM 1.5G 100 mW cm^−2^ illumination

Devices	*V*_OC_ (V)	*J*_SC_ (mA cm^−2^)	FF (%)	PCE^a^ (%)	*J*_int_^b^ (mA cm^−2^)
PPHJ	0.830	25.85	75.45	16.19 (15.80 ± 0.56)	24.94
PiPPHJ	0.836	26.61	77.57	17.26 (17.14 ± 0.22)	25.86

^a^Average values are obtained from 15 devices

^b^Calculated from the integral of EQE curves

To clarify the improvement of device performance, the carrier dynamics and recombination behavior were investigated. The *J*_*ph *_*–V*_*eff*_ plots showed that the PiPPHJ-based devices had a higher exciton dissociation probability (P) (98.29%) than the PPHJ-based devices (96.45%), indicating more effective exciton dissociation and charge extraction (Fig. 2d and Table 2) [28]. The light intensity dependent *J*_*SC*_ was used to characterize the non-geminate bimolecular recombination, and it was found that the PiPPHJ-based device had a larger α value, which suggests less bimolecular recombination (Fig. 2e). Furthermore, the dependence of *V*_*OC*_ on light intensity showed that the PiPPHJ-based device with a smaller value exhibited less trap-assisted recombination than the PPHJ-based device (Fig. S2). In addition, the PiPPHJ-based devices exhibit higher and more balanced carrier mobilities than the PPHJ-based devices (Fig. 2f), which should be attributed to the effective vertical phase separation and the balanced crystallinity between donor and acceptor.

**Table 2:** Summarized device parameters of non-halogenated solvent-printed OSCs

Devices	P (%)	α	*μ*_*h*_ (cm^2^ V^−1^ s^−1^)	*μ*_*e*_ (cm^2^ V^−1^ s^−1^)	τ_1_ (ps)	n_trap_ (cm^−3^)
PPHJ	96.45	0.80	1.22 × 10^–4^	6.50 × 10^–5^	0.88	8.49 × 10^15^
PiPPHJ	98.29	0.93	1.86 × 10^–4^	1.65 × 10^–4^	0.69	4.48 × 10^15^

**Fig. 3:**
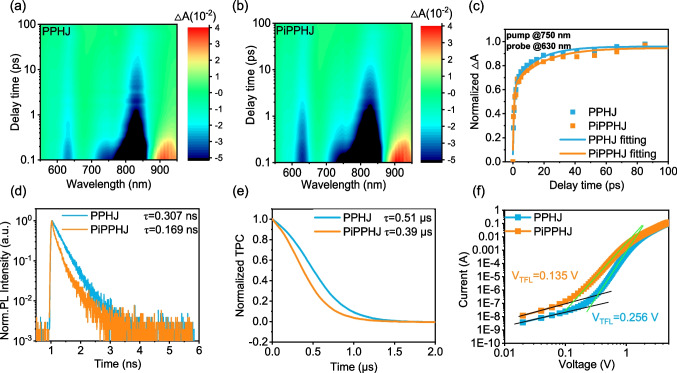
TA spectra of **a** PPHJ-based and **b** PiPPHJ-based PM6:BTP-eC9 films. **c** GSB signal results of PM6 by double exponential fitting. **d** TRPL decay curve of blend films. **e** TPC curves of the devices. **f** Trap density measurement results and current–voltage curves of electron device (solid lines are the fitting results)

To further understand the charge separation and transfer processes, the excited state dynamics was studied by transient absorption (TA) spectroscopy (Figs. 3a–c, S3). A laser beam of 750 nm was utilized to selectively excite the acceptor component. After excitation, the ground state bleaching (GSB) signal of BTP-eC9 at 850 nm appears immediately in all blend films, while the dynamics of the PM6 GSB signal (≈ 635 nm) present some differences. The hole transfer kinetics is numerically analyzed by fitting the PM6 GSB signal with the biexponential, which involves two lifetimes, i.e., the τ_1_ and τ_2_. The τ_1_ represents the ultrafast charge transfer time at the interface at the D:A interface, and the τ_2_ is assigned to the diffusion time of excitons to the interface before hole transfer. According to the data fitting and calculation, the PiPPHJ-based film exhibits smaller τ_1_ (0.69 ps) than the PPHJ-based film (0.88 ps), suggesting the fast dissociation process and efficient charge separation efficiency at the D/A interface for the PiPPHJ-based film. This conclusion is further corroborated by the time-resolved photoluminescence (TRPL) spectra (Fig. 3d), where the PiPPHJ-based film (0.169 ns) presents more effective charge transfer with the smaller lifetime than the PPHJ-based film (0.307 ns). Moreover, transient photocurrent (TPC) and transient photovoltage (TPV) were measured. As shown in Fig. 3e and S4, the photocurrent of the PiPPHJ-based device decays faster than that of the PPHJ-based device, and the carrier time of PiPPHJ-based device is longer than the PPHJ-based device. These results demonstrate that the improved photovoltaic performance of the PiPPHJ-based device is due to more effective exciton dissociation and charge extraction as well as less bimolecular and trap-assisted recombination, compared to the PPHJ-based device.

To further reveal the internal recombination behavior, the effect of trap density (n_trap_) (key semiconductor parameter) on both electron-only and hole-only devices by the space charge limit current (SCLC) method were studied (Fig. 3f and S5, Table 2) [28, 29]. Each current–voltage curve exhibits two different regions, including the ohmic region (black lines) and the trap-filling region (green lines). The hole and electron trap density were calculated by using the equation n_trap_ = 2ε_0_ε_r_V_TFL_/qL^2^, where V_TFL_, ε_0_, ε_r_, q, and L are the starting voltage of the defect filling baseline, vacuum permittivity, the relative dielectric constant, the elementary charge, and the thickness of the active layer, respectively. The PiPPHJ-based device exhibit less electron and hole defect densities (4.48 × 10^15^ cm^−3^and 1.36 × 10^17^ cm^−3^) than the PPHJ-based device (8.49 × 10^15^ cm^−3^and 1.41 × 10^17^ cm^−3^), which suppresses the charge carrier recombination.

**Fig. 4:**
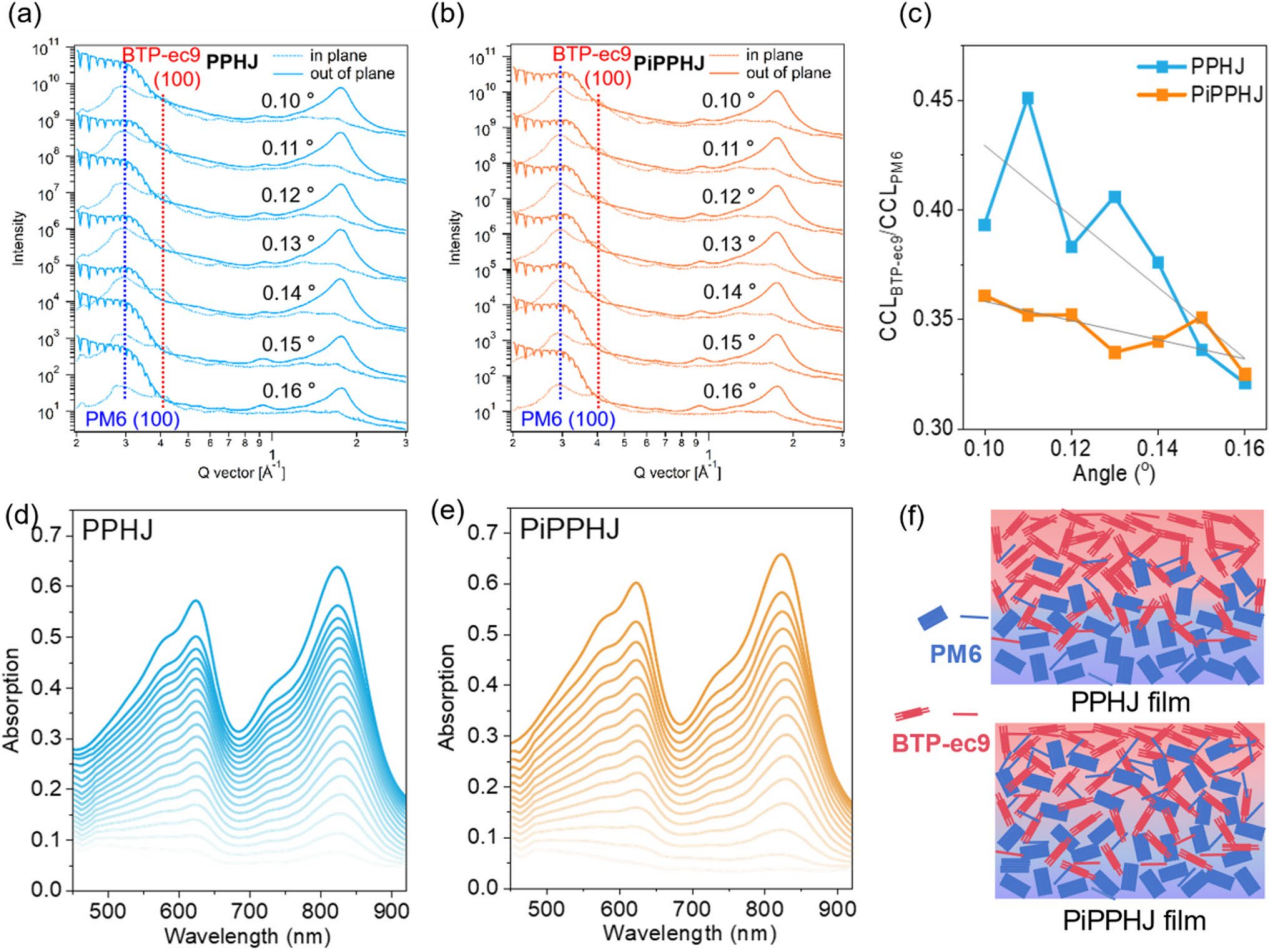
Angle-dependent GIWAXS line profiles of **a** PPHJ films and **b** PiPPHJ films. **c** CCL ratio of BTP-eC9 to PM6 at different angles. DSAS of **d** PPHJ and **e** PiPPHJ films. **f** The schematic illustration of PPHJ-based and PiPPHJ-based active layer

To clarify the above performance differences of OSCs, the vertical morphology of active layer was studied by the angle-dependent grazing incidence wide angle x-ray scattering (GIWAXS), where the molecular packing and crystallinity of films in the vertical direction can be obtained [30–32]. As show in Fig. 4a, b, the states of regions at different depths in the film are related to the grazing incidence angle (α). When α = 0.10°, the surface morphology of the film is reflected. With the increase of the incidence angle, the deep structure information of the film is gradually exposed. When the α over the critical angle of silicon substrate (0.16°), the X-ray will penetrate the entire film to the silicon substrate, thus reflecting the average structure of the whole film. The scattering peaks at q ≈ 0.29 Å^−1^ and q ≈ 0.38 Å^−1^ are corresponding to the (100) lamellar stacking peaks of PM6 and BTP-eC9, respectively. According to the Scherrer formula, the crystal coherence length (CCL) of PM6 and BTP-eC9 in the films can be calculated by fitting the (100) peaks of PM6 and BTP-eC9, which represents the crystallinity of the components. A series of CCL values with varying angles can reflect the crystalline distribution of components in the vertical direction. Thus, the angle-dependent CCL_BTP-eC9_/CCL_PM6_ values can reflect the vertical component distribution of acceptor and donor. As summarized in Fig. 4c and Table S3, the PPHJ film exhibits serious vertical component distribution, where PM6 enriched in bottom and BTP-eC9 enriched in top, resulting in insufficient D/A interfaces. However, the PiPPHJ film shows more moderate vertical component distribution, leading to more D/A mixed domains and increased D/A interface, which facilitate the exciton dissociation and improve the photovoltaic performance. Moreover, the depth-sensitive absorption-spectroscopy (DSAS) of films was also used to analyze the vertical phase separation of the active layer, which further demonstrate the favorable vertical component distribution for the PiPPHJ-based active layer (Fig. 4d, e and S6)[33]. In addition, according to the atomic force microscopy (AFM) measurements (Fig. S7), the PiPPHJ film presents uniform surface and decreased surface roughness which should be attributed to the improved processibility and the resulting high-quality film. The schematic diagrams of vertical component distribution for the active layer based on PPHJ and PiPPHJ are shown in Fig. 4f.

**Fig. 5:**
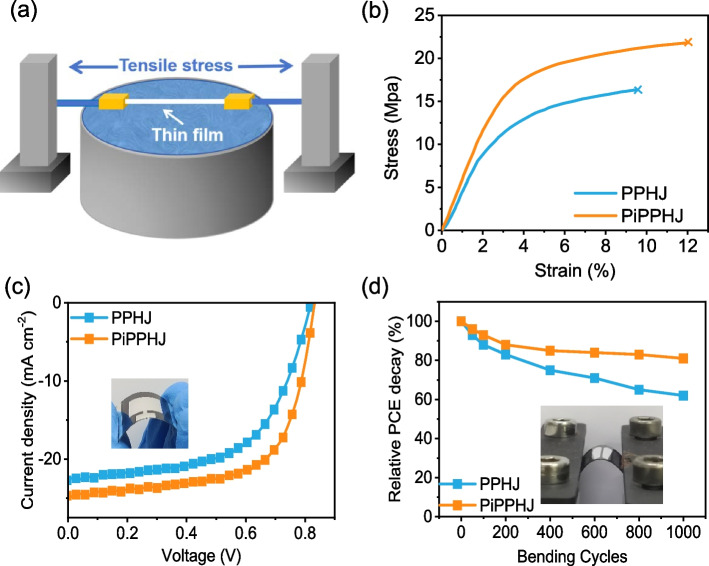
**a** The schematic illustration of tensile test equipment for the floated ultrathin film. **b** Stress–strain curves of PPHJ and PiPPHJ films. **c** The *J-V* curves of large-area (1 cm^2^) flexible devices. **d** Relative PCE decay of the PPHJ-based and PiPPHJ-based devices at bending cycles

Researches have indicated that factors such as polymer molecular weight, additives, D/A interfaces, and the morphology of active layer all influence the mechanical performance of organic electronic devices, where the mechanical flexibility is one of the key factors for the application of OSCs in wearable device [34, 35]. To accurately characterize the mechanical properties of active layers, the strain–stress curves of thin films through film on water (FOW) tensile test were performed. Figure 5a shows the schematic illustration of FOW tensile test equipment for floated ultrathin film. As displayed in Fig. 5b, the PiPPHJ-based active layer exhibits a crack onset strain of 12.0%, outperforming the PPHJ-based film (9.6%), and the tensile strength of the film increases from 16.4 to 21.8 MPa after incorporating the polymer, suggesting the better mechanical tensile flexibility of PiPPHJ. This improvement can be attributed to the chain entanglement and plasticity of the polymer. The incorporation of polymer into the acceptor layer serves to mitigate the brittle crystalline features of the small molecular layer and facilitates the creation of a more uniform contact surface. These factors collectively contribute to enhancing the mechanical strength of the film [36]. The device stability was also investigated under the continuous heating at 80 °C. As described in Fig S8, the PiPPHJ-based OSCs exhibit better device stability than the PPHJ-based OSCs. Moreover, flexible large-area (1 cm^2^) OSCs were fabricated by blade-coating and the photovoltaic performance were shown in Fig. 5c and Table S4. The PPHJ-based large-area flexible device achieves a PCE of 10.8%. However, an increased PCE of 13.3% is obtained for the PPHJ-based device. In addition, after 1000 bending cycles, the PCE of PiPPHJ-based device maintains more than 82% of the original efficiency, which outperforms the PPHJ-based device (Fig. 5d). Overall, the PiPPHJ-based device not only exhibits higher photovoltaic performance, but also have better mechanical flexibility, suggesting the effective approach of PiPPHJ strategy in non-halogenated solvent-printed organic solar cells.

## Conclusions

In summary, we introduced a PiPPHJ strategy in non-halogenated solvent-printed OSCs, involving the incorporation of a small amount of PM6 polymer into the upper BTP-eC9 acceptor layer. The addition of PM6 increased the viscosity of the acceptor solution, improved the processability of blade-coating, and effectively regulated the vertical component distribution of the active layer, promoting more D/A interfaces. As a result, PiPPHJ-based OSCs achieved an outstanding PCE of 17.26%, accompanied by a high *J*_SC_ of 26.61 mA cm^−2^ and excellent efficiency reproducibility, surpassing PPHJ-based OSCs (PCE of 16.19%, *J*_SC_ of 25.85 mA cm^−2^). Moreover, the PiPPHJ-based active layer exhibited superior tensile properties with a crack onset strain of 12.0%, outperforming the PPHJ-based film (9.6%). Additionally, we successfully fabricated large-area (1 cm^2^) flexible OSCs, achieving a remarkable PCE of 13.30% and considerable mechanical flexibility with 82% of the initial PCE after 1000 bending cycles. Overall, this approach presents a straightforward pathway to synergistically enhance processability, efficiency, reproducibility and mechanical flexibility, highlighting the substantial potential of PiPPHJ-based OSCs in flexible and wearable electronics.

### Supplementary Information

Additional file1 (DOCX 20674 KB)

## Data Availability

All data supporting the conclusions of this article are included within the article.

## List of symbols

OSCs: Organic solar cells
PPHJ: Pseudo-planar heterojunction
NFSMAs: Non-fullerene small molecule acceptors
PiPPHJ: Polymer-incorporated pseudo-planar heterojunction
PCE: Power conversion efficiency
BC: Blend-casting
SCs: Equential-casting
BHJ: Bulk heterojunction
D/A: Donor/acceptor
o-XY: O-xylene
PEDOT: PSS Poly (3,4-ethylenedioxythiophene) polystyrene sulfonate
ITO: Indium tin oxid
PDINN: *N*,*N*′-Bis{3-[3-(Dimethylamino)propylamino]propyl}perylene-3,4,9,10-tetracarboxylic diimide
PM6: Poly((4,8-bis(5- (2-ethylhexyl)-4-fluoro-2-thienyl)benzo[1,2-b:4,5-b′]dithiophene-2,6-diyl)-2,5-thiophenediyl(5,7-bis(2-ethylhexyl) -4,8-dioxo-4H,8H-benzo[1,2-c:4,5-c′]dit hiophene-1,3-diyl)- 2,5-thiophenediyl
BTP-eC9: 2,2′- [[12,13-Bis(2-butyloctyl)-12,13-dihydro-3,9-dinonylbisthieno[2″,3″:4′,5′]thieno[2′, 3′:4,5]pyrrolo[3,2-e:2′,3′-g][2,1,3]benzothiadiazole-2,10-diyl]bis[methylidyne(5,6-chlor o-3-oxo-1H-indene-2,1(3H)-diylidene) ]]bis[propanedinitrile]
EQE: External quantum efficiency
*J*_*SC*_: Short circuit current
*V*_*oc*_: Open-circuit voltage
*J-V*: Density-voltage
*J*_*ph*_: Photocurrent density
*V*_*eff*_: Effective voltage
*N*_*trap*_: Trap density
SCLC: Space-charge-limited-current
TPC: Transient photocurrent
TPV: Transient photovoltage
TRPL: Time-resolved photoluminescence
TA: Transient absorption
GIWAXS: Grazing incidence wide angle X-ray scattering
CCL: Crystal coherence length
AFM: Atomic force microscopy
FOW: Film on water
DSAS: Depth-sensitive absorption-spectroscopy
