# Supplementary material for: High-efficiency flexible organic solar cells with a polymer-incorporated pseudo-planar heterojunction
Source: Discov Nano. 2024 Mar 4;19(1):39. doi: 10.1186/s11671-024-03982-1 (PMC10912397; doi:10.1186/s11671-024-03982-1)
Supplement: Supplementary file 1 — Additional file1 (DOCX 20674 KB) [file 11671_2024_3982_MOESM1_ESM.docx]

**Supporting information**

High-Efficiency Flexible Organic Solar Cells with Polymer-Incorporated Pseudo-Planar Heterojunction

Lin Zhang^1^*· Yuxin He^1^· Wen Deng^1^· Xueliang Guo^1^· Zhaozhao Bi^2^· Jie Zeng^4^· Hui Huang^5^· Guangye Zhang^5^· Chen Xie^5^· Yong Zhang^4^· Xiaotian Hu^3^· Wei Ma^2^· Yongbo Yuan^1^· Xiaoming Yuan^1^*

**Fig. S1** *J-V* characteristics of blade-coated PM6/BTP-eC9 OSCs with various polymer content.

**Table S1** Photovoltaic parameters of blade-coated PM6/BTP-eC9 OSCs with 0.2% DIO under AM 1.5G 100 mW cm^-2^ illumination.

| polymer content | *V_oc_* [V] | *J_sc_* [mA cm^-2^] | FF [%] | PCE[%] |
| --- | --- | --- | --- | --- |
| 0% | 0.830 | 25.85 | 75.44 | 16.19 |
| 5% | 0.836 | 26.61 | 77.57 | 17.26 |
| 10% | 0.824 | 26.65 | 74.49 | 16.36 |
| 15% | 0.822 | 26.95 | 71.42 | 15.84 |

^a^ Average values are obtained from 15 devices.

**Table S2** Photovoltaic parameters of blade-coated PM6/BTP-eC9 OSCs without additives under AM 1.5G 100 mW cm^-2^ illumination.

| polymer content | *V_oc_* [V] | *J_sc_* [mA cm^-2^] | FF [%] | PCE[%] |
| --- | --- | --- | --- | --- |
| PPHJ | 0.823 | 26.00 | 72.38 | 15.50 |
| PiPPHJ | 0.834 | 26.39 | 75.00 | 16.51 |

^a^ Average values are obtained from 15 devices.

**Fig. S2** Open-circuit voltage versus light intensity plots of PPHJ-based and PiPPHJ-based.

**Fig. S3** TA spectroscopy spectra of **a** PPHJ films and **b** PiPPHJ films, **a-b** correspond to Fig. 3a-b, respectively.

**Fig. S4** TPV curves of the devices.

**Fig. S5** Trap density measurement results and Current-voltage curves of PPHJ and PiPPHJ devices, respectively. Solid lines are obtained by fitting the data.

**Table S3** The angle-dependent CCL_BTP-eC9_/CCL_PM6_ values.

| Angle (^o^) | PPHJ | | | PiPPHJ | | |
| --- | --- | --- | --- | --- | --- | --- |
|  | CCL_BTP-eC9_ | CCL_PM6_ | CCL_BTP-eC9_/CCL_PM6_ | CCL_BTP-eC9_ | CCL_PM6_ | CCL_BTP-eC9_/CCL_PM6_ |
| 0.10 | 33.27 | 84.70 | 0.393 | 35.38 | 98.07 | 0.361 |
| 0.11 | 39.37 | 87.34 | 0.451 | 35.16 | 99.82 | 0.352 |
| 0.12 | 33.47 | 87.34 | 0.383 | 35.16 | 99.82 | 0.352 |
| 0.13 | 36.06 | 88.73 | 0.406 | 34.09 | 101.63 | 0.340 |
| 0.14 | 35.61 | 94.75 | 0.376 | 34.51 | 101.63 | 0.335 |
| 0.15 | 26.12 | 77.64 | 0.336 | 32.69 | 93.17 | 0.351 |
| 0.16 | 25.29 | 78.73 | 0.321 | 27.54 | 84.70 | 0.325 |

**Fig. S6** Compositional distribution profiles at different film depths, numerically extracted from Fig.4 a and b, respectively.

**Fig. S7** AFM height images of PPHJ films and PiPPHJ films.

**Table S4** photovoltaic parameters of blade-coated flexible and Large-area OSCs with 1 cm^2^ area under AM 1.5G 100 mW cm^-2^ illumination.

| Devices | Area  [cm^2^] | *V_oc_*  [V] | *J_sc_*  [mA cm^-2^] | FF  [%] | PCE *^a^*  [%] |
| --- | --- | --- | --- | --- | --- |
| PPHJ | 1.00 | 0.82 | 22.6 | 58.23 | 10.80 |
| PiPPHJ | 1.00 | 0.83 | 24.67 | 64.80 | 13.30 |

^a^ Average values are obtained from 15 devices.

**Fig S8** The photovoltage performance of OSCs under the continuous heating at 80 ^o^C.
